# Supplementary material for: Characterization and field application of a novel dual-liquid gas leakage material: Mechanical properties and microscopic hydration mechanism
Source: PLoS One. 2023 Apr 12;18(4):e0284140. doi: 10.1371/journal.pone.0284140 (PMC10096304; doi:10.1371/journal.pone.0284140)
Supplement: Attachment — Submitted filename: Response to Reviewers.docx [file pone.0284140.s003.docx]

Dear Prof./Dr. Jianguo Wang Editor-in-Chief:

Thank you very much for your letter. All authors would like to take this opportunity to express our gratitude to your valuable comments and suggestions on (Ref: PONE-D-22-23213). Paper title: “Experimental characterization and field application of a novel dual-liquid gas leakage material: mechanical properties and microscopic hydration mechanism”.

According to your suggestions, we have carefully modified the manuscript and made it easily to be understood. We hope the revised manuscript can meet these requests and be accepted. The revised manuscript is attached here, and the questions are answered one by one in the enclosed files.

I am looking forward to receiving your decision as soon as possible.

With my best regards.

Zi-jie Hong, Jian-ping Zuo, Zhen-hua Li, Lei Xu

**Responds to the academic editor’s comments:**

**Comment 1:** As an experimental research on gas drainage, is it necessary to introduce a simple parameter introduction (in the form of pictures or text descriptions) to the overall storage model or prototype site? It seems that there is no relevant content in the draft.

**Response:** Thank you for your valuable advice. **According to the comment, the author adds the field parameter.**

**Comment 2:** As a physical experiment, the verification of accuracy (or how to avoid experimental errors) is a very important part. Should the authors add the verification content into the manuscript?

**Response:** Thank you for your valuable advice. **The results obtained by the author are focused on the mechanical properties of the sealing materials, which can be used to meet the requirements of field tests. According to the requirements, the author completed the field test, and achieved good results. The correctness of the experiment is verified.**

**Comment 3:** The picture quality in the manuscript is so poor that the details of the picture and the labels on the picture are hard to see (such as SEM pictures). However, it is clearer in the response section on the contrary, the author should attach pictures with higher-quality versions in the text.

**Response:** Thank you for your valuable advice. **The problem of picture quality may be due to the system's processing of pictures. All pictures should be processed by the system when submitting, otherwise the submission cannot be completed. Meanwhile, according to the comment, the author revised some of the pictures.**

**Comment 4:** It is recommended to polish the overall text to make the expression more reasonable.

**Response:** Thank you for your valuable and thoughtful comments. **The author used a firm called MogoEdit to complete the revision. The proof of modification is as follows:**

**Comment 5:** If the pictures are listed separately, it is recommended to add the titles below the pictures instead of leaving the titles in the text.

**Response:** Thank you for your valuable comments. **The journal required that the picture and the title should be separated, thus the author treated it according to the author guidance.**
